# On the coactions between perceived program quality and youth purpose: Toward a model of dynamic person⇔context relations

**DOI:** 10.1111/jora.70277

**Published:** 2026-10-06

**Authors:** Ruoying Zhang, Roya Abbasi‐Asl, Margaret Mackin, Jacqueline V. Lerner, Richard M. Lerner, Alistair Sim, Elizabeth M. Dowling, Jonathan M. Tirrell, Natasha Keces, Alexa Hasse, Kirsten Olander, Kiana Douglas, Patience Kibbedi, Jane Wanyama

**Affiliations:** ^1^ Lynch School of Education and Human Development Boston College Chestnut Hill Massachusetts USA; ^2^ Eliot‐Pearson Department of Child Study and Human Development Tufts University Medford Massachusetts USA; ^3^ Compassion International Colorado Springs Colorado USA; ^4^ Compassion International Kampala Uganda

**Keywords:** Big Three, Compassion International Study of PYD, perceived program quality, positive youth development, purpose, Uganda

## Abstract

Contemporary theories of positive youth development (PYD) emphasize dynamic, mutually beneficial relationships between individuals and their contexts as a pathway to thriving and contribution. However, there remains a critical gap in longitudinal, theory‐driven research examining youth strengths (e.g., purpose) and contextual assets (e.g., program quality) in Majority World contexts, particularly within structured youth programming. The present study examined reciprocal relationships between perceived program quality (a higher‐order factor of the Big Three) and youth purpose using three waves of longitudinal data from a sample of 615 adolescents (50.3% female; ages 9–16, *M*_age_ = 12.5, *SD*_age_ = 2.3) participating in Compassion International youth development programs in Uganda. Results using cross‐lagged panel models revealed positive, reciprocal associations between perceived program quality and youth purpose, such that higher program quality predicted subsequent higher youth purpose, and stronger youth purpose predicted more positive later perceptions of program quality. Temporal precedence tests indicated that neither construct emerged as a dominant developmental driver, and therefore supported a pattern of mutual influence. These findings advance PYD theory by providing longitudinal evidence for dynamic person⇔context coactions between perceived program quality and youth purpose, consistent with dynamic, relational developmental systems perspectives.

## INTRODUCTION

Youth development progresses through ongoing coactions between young people and the contexts in which they grow. Within the positive youth development (PYD) framework, structured youth programs represent critical developmental settings that can support youth strengths, particularly when program quality is experienced as meaningful and responsive (Arnold & Ferrari, 2025; Gootman & Eccles, 2002; Lerner et al., 2015). At the same time, internal strengths such as youth purpose may shape how young people engage with and interpret these program environments, suggesting the potential for dynamic, bidirectional relations between youth and contexts (Damon, 2008; Lerner et al., 2015). Despite growing interest in program effectiveness, relatively few longitudinal studies have examined these processes in Majority World contexts. Situated within the Compassion International (CI) Study of PYD in Uganda, the present study addresses this gap by examining the longitudinal and reciprocal relations between perceived program quality and youth purpose.

### Perceived program quality: Big Three features of effective youth programs

Contemporary theories of human development conceptualize development as emerging from ongoing, reciprocal relations between individuals and their contexts (Lerner, 2026; Overton, 2015). From a dynamic, relational developmental systems (RDS) perspective, individuals are viewed as active, self‐organizing, and self‐regulating agents whose development emerges through dynamic, nonlinear, and complex coactions across multiple levels of organization over time (Lerner, 2026). Accordingly, development is not driven solely by individual characteristics or environmental inputs, but by their reciprocal, mutually influential relations—often represented by a bidirectional arrow (⇔), which emphasizes the dynamic and coacting nature of person⇔context processes (Overton, 2015). When these individual⇔context coactions are mutually beneficial and developmentally adaptive, they promote positive development and evolve over historical time (Elder Jr. et al., 2015; Lerner, 2026). Within the PYD framework, thriving is expected to occur when youth strengths are aligned with contextual assets in ways that are mutually reinforcing and sustained across development (Lerner et al., 2015).

Youth development programs represent a contextual asset in which such person⇔context coactions may unfold. Rather than functioning as static environments, these programs operate as dynamic developmental settings that are experienced by youth through sustained engagement, interpretation, and coaction, making youth's perceptions of program quality an essential component of the developmental process (see Spencer, 2006). One influential conceptualization of effective developmental contexts within youth programming is captured by the Big Three features of effective youth programs: (1) positive and sustained adult–youth relationships, (2) life‐skill‐building activities, and (3) youth contribution and leadership opportunities (Lerner, 2004; Tirrell et al., 2020). These features are theorized to operate within safe environments to support youth engagement, motivation, and growth.

Importantly, within a dynamic person⇔context framework, the Big Three are not merely program characteristics functioning in a unidirectional manner. Instead, they reflect contextual assets whose developmental significance may depend on how youth engage with, respond to, and shape their program experiences over time. Youth who bring different strengths, goals, and orientations into program contexts may differentially perceive and utilize these features, suggesting the potential for reciprocal relations between perceived program quality and youth strengths (Spencer, 2006; Tirrell et al., 2020). However, empirical research has rarely examined the Big Three within longitudinal designs that can capture such dynamic coactions.

Most research on perceived program quality has been qualitative, and mostly conducted in the U.S. context (DeSouza, 2017; Hershberg et al., 2015). In the international context, and specifically within Majority World countries, Tirrell et al. (2020) developed and validated the Big Three measure using data from the CI Study of PYD in Rwanda, providing initial evidence for its reliability, validity, and measurement invariance. Building upon this study, subsequent work in Rwanda demonstrated that the Big Three measure functioned as a meaningful representation of contextual assets in a national context other than the United States, with perceived program quality predicting youth strengths and relating to youth contribution, consistent with prior findings from Rwanda (Tirrell et al., 2021). Despite these advances, the extent to which perceived program quality, as captured by the Big Three, demonstrates longitudinal stability and dynamic relations with youth strengths across multiple waves and within additional national contexts, such as Uganda, remains unexamined.

### Perceived program quality and youth purpose

Purpose in life refers to an intraindividually stable, forward‐looking intention to pursue goals that are personally meaningful and oriented toward contributing beyond the self (Damon, 2025; Damon et al., 2019). In adolescence, purpose plays an important role by providing direction for long‐term goal pursuit, becoming integrated into the developing identities of youth, and motivating action toward making a positive difference in the world. Empirical research distinguishes between purpose identification, defined by clarity and commitment to meaningful aims, and purpose searching, which involves ongoing exploration of goals, values, and sources of motivation (Bronk et al., 2009; Steger & Kashdan, 2007). Although analytically distinct, these processes are often iterative and dynamically interrelated across development, particularly during periods of heightened contextual influence, such as participation in structured youth programs (Bronk, 2014).

The important role of these structured environments is well supported by a broader literature emphasizing the relational nature of purpose development (Bronk, 2024; Damon, 2025). For example, Liang and colleagues highlight that purpose is rarely developed in isolation; rather, it is dynamically cultivated through supportive social contexts and high‐quality adult–youth interactions (Liang et al., 2017). According to this framework, it is the quality of these relational connections—such as effective mentoring and adult support—that provides the essential social scaffolding for youth to explore their values (Liang et al., 2017; Lund et al., 2019). Through these high‐quality program interactions, young people are better equipped to transform abstract aspirations into an actionable, prosocial “purpose mindset” (Liang & Klein, 2022). Consequently, how youth perceive the quality of support and engagement within their programs serves as a vital relational process for facilitating purpose development (Spencer, 2006).

In addition, the development of purpose is deeply contextualized by a young person's environment, particularly for youth navigating under‐resourced backgrounds. Whereas economic and systemic hardships are sometimes framed as barriers to thriving, recent empirical work highlights a more complex reality. Youth from low‐income communities are just as likely to report leading lives of purpose as their middle‐income peers, utilizing purpose as a critical psychological asset to navigate their environments and achieve positive developmental outcomes (Bronk, 2024; Bronk et al., 2020). Psychological stress stemming from financial, neighborhood, or academic challenges can initially impede purpose exploration by leaving youth feeling overwhelmed; however, when coupled with strong social and familial support, these same stressors frequently act as powerful motivators (Gutowski et al., 2018).

A growing body of research suggests that purpose can function as a central organizing strength that supports the development of other character attributes, including gratitude, forgiveness, contribution, and generosity, and promotes both individual and community well‐being (Abbasi‐Asl, Yu, Hasse, et al., 2025; Barcaccia et al., 2023; Tirrell et al., 2025; see also Damon, 2008, 2025). Purpose has been linked to greater hope, optimism, and life satisfaction by fostering meaningful goals and perceived agency in their pursuit (Bronk, 2024; Bronk et al., 2009; Damon, 2025; Snyder et al., 1997). Purpose is also associated with academic engagement and persistence, as well as with resilience, grit, and a stronger sense of meaning in school‐related activities (Hill et al., 2016; Yeager & Bundick, 2009). Longitudinal evidence suggests that purpose is related to long‐term physical health and well‐being, including reduced cognitive decline and increased longevity (Boyle et al., 2009; Damon, 2025; Hill et al., 2016). Collectively, these findings position purpose as a key internal strength that supports both personal thriving and contribution to the broader community.

From a dynamic, RDS perspective, purpose does not develop in isolation. Rather, it emerges through ongoing person⇔context relations, in which supportive developmental contexts play a critical role in shaping youths' opportunities to explore, articulate, and enact meaningful goals (Damon, 2025; Damon et al., 2019; Lerner, 2026; Overton, 2015). High‐quality youth programs may be especially well positioned to foster purpose by providing consistent relationships with caring adults, opportunities for skill‐building and reflection, and contexts that encourage contribution and leadership (Abbasi‐Asl, Yu, Tirrell, et al., 2025). When youth perceive programs as supportive, engaging, and responsive to their needs, these contexts may amplify the sense of direction among youth, reinforce purpose‐related identities, and translate emerging aspirations into sustained action.

At the same time, youth purpose may actively shape how they engage with these contexts and how program features are perceived (Bronk, 2014; Damon et al., 2019). Youth with clearer or more strongly held purposes may participate more intentionally, seek out relational and leadership opportunities, and interpret program experiences as more meaningful and supportive (Bronk, 2024; Bronk, 2014; Bundick et al., 2010). Through these reciprocal processes, perceived program quality and youth purpose may mutually reinforce one another over time, positioning program contexts not only as catalysts for purpose development but also as systems that are dynamically shaped by youths' purposive engagement.

### Compassion International Study of positive youth development in Uganda

The present study is situated within the CI Study of PYD, a longitudinal, multi‐nation research initiative and research–practitioner collaboration designed to evaluate how CI youth development programs function as developmental contexts for children and adolescents living in poverty (Compassion International, n.d.‐a).

CI began its work in Uganda in 1980 and currently serves more than 140,000 children and adolescents through partnerships with hundreds of local churches (Compassion International, n.d.‐b). Consistent with CI's emphasis on individualized care and PYD principles, the CI Study of PYD in Uganda has prioritized person‐specific and longitudinal approaches to assessment. This program foundation has enabled the present study to examine perceived program quality and youth purpose as dynamically related components of youth development within a culturally and structurally specific context.

Uganda has one of the youngest population profiles globally (Uganda Bureau of Statistics [UBoS], 2024). Many Ugandan youth, however, face substantial challenges such as economic hardship, limited access to health services, and exposure to environmental stressors. Empirical work has linked these conditions to elevated risks for substance use, emotional distress, and compromised quality of life among Ugandan youth (Culbreth et al., 2021; Olema et al., 2014). Exposure to violence in family and community settings further increases the likelihood of internalizing symptoms, trauma‐related difficulties, and maladaptive coping behaviors (Kaggwa et al., 2022). These risks are intensified by broader structural challenges, including high burdens of infectious disease and substantial barriers to accessing mental health care, particularly in rural and under‐resourced areas (Kigozi et al., 2010).

In response to these challenges, scholars, and practitioners increasingly emphasize the importance of strengths‐based approaches to youth development that move beyond deficit‐oriented models (Lerner et al. 2021). Within the PYD framework, youth are viewed as active agents in their own development, possessing strengths that can be nurtured through supportive developmental contexts (Lerner, 2026). Strengths‐based programs are particularly relevant in Uganda, where structural adversity may limit access to material resources but does not diminish youth potential for positive change. Programs that intentionally cultivate character, self‐regulation, and meaning‐making may serve as significant contextual assets that support adaptive functioning and resilience among youth facing cumulative risk (Berkowitz, 2012; Berkowitz & Bier, 2024).

Within this context, purpose is a particularly salient developmental strength for youth. Purpose provides youth with a sense of meaning, direction, and commitment to goals that extend beyond the self, which may be especially important for navigating adversity and uncertainty (Burrow & Hill, 2011; Damon et al., 2019). Prior research in Uganda supports the positive association of youth purpose with a wide range of other character attributes, including spirituality, gratitude, forgiveness, contribution, and generosity, and with well‐being (Abbasi‐Asl, Yu, Hasse, et al., 2025; Abbasi‐Asl, Yu, Tirrell, et al., 2025; Tirrell et al., 2025). In contexts characterized by economic hardship and social instability, purpose may help youth interpret challenges as meaningful and sustain engagement in prosocial and future‐oriented pathways. In these structurally constrained contexts, and indeed in all contexts of youth development, our RDS‐based conceptual orientation suggests that idiographic development is part of the ontogenetic pathway of all youth. Therefore, for some youth, purpose is an already present strength, whereas for other youth it is a strength that must be developed (e.g., through character education; Berkowitz, 2021). Nevertheless, for both instances of youth development, the ontogenetic presence of purpose can only be sustained or developed as part of a dynamic developmental process that requires significant, intentional support from others across time and place. Thus, without such contextual support, purpose must be understood as a developmental asset for all youth (Damon, 2025) that can be compromised (e.g., diminished or prevented from adequately developing) in the face of contextual disruption precipitated by economical, educational, health, environmental, or political hardships besetting under‐resourced populations (Gutowski et al., 2018; Lerner, 2026; Spencer & Dowd, 2024). Whereas purpose ultimately functions as a vital psychological asset once established, the systemic barriers that may be faced by youth, such as those from Uganda who were studied in the present research, mean that the process of identifying and committing to meaningful goals requires intentional, robust support across ontogenetic time and place. Consequently, structured developmental contexts can become critical compensatory environments that mitigate the disruptive effects of structural constraints. However, little is known about how purpose is associated with structured developmental contexts such as youth programs among Ugandan youth.

### The present study

The present study advances research on perceived program quality and youth development by examining longitudinal and reciprocal relations between perceived program quality, as captured by the Big Three features of effective youth programs, and youth purpose among Ugandan adolescents participating in CI youth development programs.

Specifically, the study pursues two main goals. First, it investigates reciprocal relations between perceived program quality and youth purpose over time using a cross‐lagged panel modeling approach. Second, it tests temporal precedence to determine whether perceived program quality predicts subsequent changes in youth purpose, whether youth purpose predicts changes in perceived program quality, or whether relations are bidirectional.

## METHOD

### Participants

The current study included 615 Ugandan adolescents (50.3% female; ages 9–16, *M*_age_ = 12.5, *SD*_age_ = 2.3) across three waves of data collection. Data were collected across 24 project sites of CI, with the majority (84%) of participants residing in rural areas. At Wave 1, the sample consisted of 606 youth (*M*_age_ = 12.77, *SD*_age_ = 2.33), of whom 304 were female (50.2%). Most participants resided in rural areas (*n* = 509, 84.0%), with smaller proportions living in urban areas (*n* = 49, 8.1%) and semi‐urban or semi‐rural areas (*n* = 48, 7.9%). At Wave 2, the sample included 591 youth (*M*_age_ = 13.91 years, *SD*_age_ = 2.36), with 297 females (50.3%). One participant was newly enrolled at Wave 2. The majority of participants continued to reside in rural areas (*n* = 495, 83.8%), followed by urban areas (*n* = 54, 9.1%) and semi‐urban or semi‐rural areas (*n* = 42, 7.1%). At Wave 3, the sample comprised 576 youth (*M*_age_ = 14.93 years, *SD*_age_ = 2.34), including 290 females (50.3%). Eight participants were newly enrolled at Wave 3. Most participants lived in rural areas (*n* = 482, 83.7%), with smaller proportions residing in urban (*n* = 62, 10.8%) and semi‐urban or semi‐rural areas (*n* = 32, 5.6%).

### Procedure

Data for the present study were drawn from the CI Study of PYD in Uganda, a three‐wave longitudinal study spanning from April 2022 to September 2024. Wave 1 data collection took place from April to May 2022. Wave 2 data collection occurred in August and September 2023, and Wave 3 data collection occurred in August and September 2024. At each wave, trained data collectors administered the survey in a one‐on‐one interview format, reading items aloud and recording participants' responses using tablet‐based online surveys. Surveys took approximately 30–45 min to complete and included items beyond the purpose of the current study.

CI national staff in Uganda selected 24 project sites from both urban and rural regions for participation in the study. Sites were chosen based on program implementation considerations and indicators of strong program functioning (e.g., graduation rates and youth participation in program‐related activities). Following institutional ethical approval and the acquisition of informed consent, youth enrolled in CI‐supported programs were recruited for participation.

Data collectors were recruited locally, primarily from a nearby university or based on prior experience working with children and digital data collection tools. All data collectors completed a three‐day training program prior to data collection. Training included an overview of CI's mission and child protection policies, instruction on study goals and survey content, and hands‐on practice with survey administration procedures. On the final day of training, data collectors piloted the survey with CI‐supported youth to assess clarity, cultural appropriateness, and respondent burden. Feedback from these pilot sessions was documented and used to make final adjustments to the survey.

All study materials were translated from English into five local languages commonly used at the participating project sites—Ateso, Lumasaba, ruKiga, ruNyankole, and ruTooro—and then back‐translated into English to ensure translation accuracy. Surveys were administered in a flexible combination of English and local languages based on participants' comfort and preference. As a result, interview administration varied slightly across participants with respect to language use, although the content and structure of the survey remained consistent. Participating sites received nonmonetary tokens of appreciation, such as gift baskets, shoes, or clothing, as determined by CI project staff.

### Measures

#### Perceived program quality

Subscales used to assess youth perceptions of program quality were drawn from the Tirrell et al. (2020) measures of the Big Three features of effective youth programs, including (1) positive and sustained adult–youth relationships, (2) life‐skill‐building activities, and (3) opportunities for leadership and contribution. For each subscale, participants indicated their level of agreement with a series of statements using a visual analog interval scale ranging from 0 (not at all) to 10 (completely agree).

##### Positive and sustained adult–youth relationships

Seven items were used to assess youth perceptions of having caring and supportive adults at the youth program. Youth indicated, from “Not at all” to “Completely” how much adults at the church project do the following? A sample item is: “Care for you.” Using the Wave 1 data set, initial CFA analyses were conducted, which resulted in the removal of one item (“Help you to gain the skills you need to succeed in life.”). The modification indices indicated a large residual covariance between this item and another item in the “Life‐Skill‐Building Activities” subscale (“Learn skills that help you succeed in life”) (Modification Index = 84.616, Expected Parameter Change = 0.517), suggesting redundancy in measurement. The analyses in the current study build on this initial CFA model (see Appendix S1: Initial CFA Model Specification and Item Modification). The six remaining items had McDonald's omega coefficients of .90 at Time 1, .89 at Time 2, and .90 at Time 3.

##### Life‐skill‐building activities

Six items were used to assess youth perceptions of learning skills at the youth program that might help them to succeed in life, such as solving problems, overcoming challenges, and getting along with others. Youth indicated, from “Not at all” to “Completely” how much the church project does the following? A sample item is: “Learn how to solve problems.” The six items had McDonald's omega coefficients of .85 at Time 1, .88 at Time 2, and .90 at Time 3.

##### Opportunities for participation in and leadership of valued activities

This component in the original measure of the Big Three, developed by Tirrell et al. (2020), included seven items assessing youth perceptions of their participation in and leadership of valued program activities. During the pilot phase of the Big Three measure in Uganda, one item was removed based on feedback from participants and data collectors. Accordingly, six items were administered in the present study. Youth indicated, from “Completely disagree” to “Completely agree,” how much do you agree or disagree with the following statements? A sample item is: “You make meaningful contributions to the church project.” The six items had McDonald's omega coefficients of .83 at Time 1, .88 at Time 2, and .90 at Time 3.

#### Youth purpose

Seven items were used to assess youths' sense of purpose. Items were derived from the Claremont Purpose Scale (Bronk et al., 2018) and culturally adapted to fit the Ugandan context, with the aim of capturing youth perceptions of meaningfulness, goal orientation, and purpose beyond the self. Youth indicated, from “Completely disagree” to “Completely agree,” how much they agreed or disagreed with a series of statements assessing purposeful thoughts and orientations. A sample item is: “In the past week, you thought about how you want to contribute to your world, family, or community.” Responses were provided on an 11‐point scale ranging from 0 to 10. The seven items demonstrated acceptable internal consistency, with McDonald's omega coefficients of .89 at Time 1, .88 at Time 2, and .88 at Time 3.

### Analysis plan

To address the research questions, a series of cross‐lagged panel models (CLPMs) were estimated in Mplus 8.8 (Muthén & Muthén, 1998–2017). CLPMs allow for the assessment of rank‐order stability and reciprocal associations among constructs over time. Although alternative longitudinal approaches (e.g., CLPMs with random intercepts; see Hamaker et al., 2015) can model within‐person change, the present study included only three measurement occasions spaced approximately 1 year apart. With only three waves, these models are not suitable for reliably estimating stable trait components or within‐person fluctuations. Therefore, the focus of the current analyses was on rank‐order change and temporal associations over time. To account for the nested structure of the data (participants clustered within 24 program sites), all models were estimated using the TYPE = COMPLEX command in Mplus. Standard errors and chi‐square test statistics were adjusted using the robust maximum likelihood (MLR) estimator to correct for the nonindependence of observations within sites.

Before conducting the main analyses using CLPMs, longitudinal measurement invariance analyses were conducted to ensure that perceived program quality and youth purpose were measured equivalently over time. Specifically, longitudinal measurement invariance was tested for the three first‐order Big Three factors of perceived program quality and youth purpose across Waves 1–3 using confirmatory factor analysis in Mplus. Configural, weak (metric), and strong (scalar) invariance models were estimated sequentially. No additional covariates were included; however, to account for the repeated‐measures design, residual variances for identical items were allowed to covary across all three waves.

Then, to examine the associations between perceived program quality and youth purpose over time, perceived program quality was modeled as a second‐order latent construct comprising the Big Three features of effective youth programming, and youth purpose was modeled as a latent construct indicated by its respective items.

An initial unconstrained CLPM was estimated with (a) autoregressive paths for each construct, (b) cross‐lagged paths between constructs, and (c) freely estimated within‐time covariances at Wave 1 and residual covariances at subsequent waves. To test whether paths were stable across the three waves, sets of equality constraints were sequentially imposed. Autoregressive paths for purpose and perceived program quality were constrained first, followed by constraints on the cross‐lagged paths and then on within‐time correlations. To evaluate measurement invariance, the fit of each progressively constrained model was compared to its less‐constrained counterpart. The change in the Comparative Fit Index (ΔCFI), and the change in the Standardized Root Mean Square Residual (ΔSRMR) were considered when comparing models. Following established methodological guidelines (Chen, 2007; Cheung & Rensvold, 2002; Kline, 2023), equivalent fit was supported if the ΔCFI was ≤ 0.010, alongside a ΔSRMR ≤ 0.030 for metric invariance and ≤ 0.015 for scalar invariance. Constraints were retained when this combination of indices indicated that the measurement model did not significantly worsen.

Further analyses were conducted to determine whether perceived program quality or youth purpose exhibited temporal precedence. Within the CLPM framework, temporal precedence is defined as the extent to which rank‐order change in one construct is associated with later rank‐order change in another construct, above and beyond the influence in the opposite direction (Shadish et al., 2002). The magnitudes of the reciprocal cross‐lagged paths were compared. A model in which the cross‐lagged paths (perceived program quality → youth purpose and youth purpose → perceived program quality) were constrained to be equal was compared to the final model from the first series of analyses. If model fit did not significantly worsen, the constrained model was considered statistically equivalent, indicating no temporal precedence. If fit decreased significantly, the relative magnitudes of the two cross‐lagged coefficients would be compared to determine directionality.

Model fit for all models was evaluated using commonly recommended indices: the Comparative Fit Index (CFI), Tucker–Lewis Index (TLI), Root Mean Square Error of Approximation (RMSEA) with 90% confidence intervals, and the Standardized Root Mean Square Residual (SRMR). Following Hu and Bentler (1999), RMSEA and SRMR values < 0.05 were considered indicative of good fit (<0.08 acceptable), and CFI/TLI values ≥0.90 acceptable (≥0.95 good).

## RESULTS

### Longitudinal invariance testing: Perceived program quality

As shown in Table 1, results indicated that strong (scalar) invariance was established across all three waves for the Big Three features of effective youth programming. The configural model demonstrated good fit, indicating that the overall factor structure was consistent over time. Constraining factor loadings to equality (weak invariance) resulted in negligible changes in model fit (ΔCFI = 0.001; ΔSRMR = 0.012), supporting metric invariance. Further constraining item intercepts (strong invariance) also resulted in acceptable model fit (ΔCFI = 0.004; ΔSRMR = 0.002), supporting scalar invariance.

**TABLE 1:** Model fit statistics for longitudinal invariance tests of perceived program quality and purpose among Ugandan youth.

Model fit statistics for longitudinal invariance tests for waves 1–3 of the measurement model of perceived program quality								
Model	*χ* ^2^(*df*)	*p*	RMSEA [90% CI]	SRMR	CFI	TLI	ΔCFI	ΔSRMR
1. Configural	1829.200* (1287)	.0000	0.026 [0.023, 0.029]	0.037	0.956	0.952		
2. Weak/loading	1877.259* (1317)	.0000	0.026 [0.024, 0.029]	0.049	0.955	0.951	0.001	0.012
3. Strong/intercept	1957.529* (1347)	.0000	0.027 [0.024, 0.030]	0.051	0.951	0.948	0.004	0.002

Model fit statistics for longitudinal invariance tests for waves 1–3 of the measurement model of youth purpose								
Model	*χ* ^2^ (*df*)	*p*	RMSEA [90% CI]	SRMR	CFI	TLI	ΔCFI	ΔSRMR
1. Configural	311.881* (165)	.0000	0.038 [0.032, 0.044]	0.037	0.960	0.949		
2. Weak/loading	324.713* (177)	.0000	0.037 [0.030, 0.043]	0.042	0.960	0.952	0.000	0.005
3. Strong/intercept	354.282* (189)	.0000	0.038 [0.032, 0.044]	0.046	0.955	0.950	0.005	0.004

*Note*: *x2* = Chi‐square; *df* = degrees of freedom; *RMSEA* = root mean square error of approximation; *CI* = confidence interval; *SRMR* = standardized root mean square residual; *CFI* = comparative fit index; *TLI* = Tucker‐Lewis index.*p < .001.

These results indicated that observed changes and longitudinal associations involving perceived program quality reflect true developmental or relational changes rather than measurement artifacts, thereby justifying the use of latent variables in subsequent longitudinal analyses.

To confirm the appropriateness of modeling perceived program quality as a higher‐order construct, we compared the fit of the higher‐order model to a first‐order correlated factors model. Because the models were estimated using MLR, a Satorra‐Bentler scaled chi‐square difference test was conducted. The less‐constrained first‐order model yielded a fit of *χ* ^2^(1401) = 2044.28, whereas the higher‐order model yielded a fit of *χ* ^2^(1433) = 2117.73. Although the scaled chi‐square difference test was statistically significant (Δ*χ* ^2^(32) = 63.36, *p* < .001), changes in practical fit indices were negligible (ΔCFI = 0.003, ΔRMSEA = 0.001). Given this equivalence in practical fit, the higher‐order model was retained for all subsequent longitudinal analyses. This approach is more parsimonious, mitigates potential collinearity among the first‐order sub‐dimensions, and best aligns with theoretical frameworks that conceptualize perceived program quality as a holistic, integrated developmental experience.

Standardized model results indicated that the measurement model for perceived program quality fit the data exceptionally well. All observed survey items loaded significantly and positively onto their respective first‐order latent factors across all three time points, with standardized factor loadings ranging from .50 to .80 at Wave 1, .58 to .81 at Wave 2, and .69 to .85 at Wave 3 (all *p*s < .001). Furthermore, demonstrating strong support for the higher‐order structure, these first‐order factors loaded highly and significantly onto the second‐order “Big Three” factor. Standardized second‐order factor loadings were robust across time, ranging from .79 to .97 at Wave 1, .80 to .94 at Wave 2, and .80 to 1.00 at Wave 3 (all *p*s < .001).

### Longitudinal invariance testing: youth purpose

As shown in Table 1, results supported configural and weak (metric) invariance for the youth purpose measure across all three waves. The configural model demonstrated good overall fit, indicating a consistent factor structure over time (CFI = 0.960, RMSEA = 0.038). Constraining factor loadings to equality across waves resulted in no meaningful decrement in model fit (ΔCFI = 0.000; ΔSRMR = 0.005), supporting metric invariance. Further constraining item intercepts (strong invariance) also resulted in acceptable model fit (ΔCFI = 0.005; ΔSRMR = 0.004), supporting scalar invariance. These results indicated that observed changes and longitudinal associations involving youth purpose reflect true developmental or relational changes rather than measurement artifacts, thereby justifying the use of latent variables in subsequent longitudinal analyses.

Standardized model results indicated a strong measurement model for the youth purpose construct. All observed survey items loaded significantly and positively onto their respective latent purpose factor across all three waves. Demonstrating robust measurement over time, the standardized item‐level factor loadings ranged from .70 to .77 at Wave 1, .66 to .77 at Wave 2, and .60 to .79 at Wave 3 (all *p*s < .001).

### Relations between perceived program quality and youth purpose over time

Fit indices for the baseline and sequentially constrained CLPMs are presented in Table 2. The initial unconstrained model (Model 1) demonstrated excellent fit to the data (CFI = 0.931, TLI = 0.928, RMSEA = 0.029 [90% CI: 0.027, 0.030], SRMR = 0.055).

**TABLE 2:** Model fit indices of baseline model and temporal precedence testing.

Model	Description	# free params	*χ* ^2^ (*df*)	Δ*χ* ^2^ (Δ*df*)	CFI	TLI	RMSEA [90% CI]	SRMR
1	Unconstrained (baseline CLPM)	249	4003.817 (2676)	—	0.931	0.928	0.029 [0.027, 0.030]	0.055
2	Constrain youth purpose autoregressive paths	248	4004.674 (2677)	0.857 (1)	0.931	0.928	0.029 [0.027, 0.030]	0.055
3	Constrain perceived program quality autoregressive paths	247	4005.305 (2678)	0.631 (1)	0.931	0.928	0.029 [0.027, 0.030]	0.055
4	Constrain cross‐lagged perceived program quality → purpose	246	4006.213 (2679)	0.908 (1)	0.931	0.928	0.029 [0.027, 0.030]	0.055
5	Constrain cross‐lagged purpose → perceived program quality	245	4011.750 (2580)	5.537 (1)	0.931	0.928	0.029 [0.027, 0.030]	0.056
6	Final selected baseline CLPM	243	4021.963 (2682)	10.213 (2)	0.930	0.928	0.029 [0.027, 0.030]	0.060
7	Temporal precedence model	242	4025.414 (2683)	3.451 (1)	0.930	0.928	0.029 [0.027, 0.030]	0.063

*Note*: Model 1 = Unconstrained baseline cross‐lagged panel model (CLPM) including youth purpose and perceived program quality. Model 2 = CLPM with autoregressive paths for youth purpose constrained to be equal across adjacent waves. Model 3 = CLPM with autoregressive paths for perceived program quality constrained to be equal across adjacent waves. Model 4 = CLPM with cross‐lagged paths from perceived program quality to youth purpose constrained to equality across waves. Model 5 = CLPM with cross‐lagged paths from youth purpose to perceived program quality constrained to equality across waves. Model 6 = Final selected baseline CLPM with additional equality constraints imposed on within‐time correlations across waves. Model 7 = CLPM in which cross‐lagged paths between youth purpose and perceived program quality are constrained to be equal across directions and waves, serving as a test of temporal precedence.

Autoregressive paths for perceived program quality and youth purpose were invariant across adjacent waves, as imposing equality constraints did not significantly worsen model fit (Models 2–3). Similarly, cross‐lagged paths from perceived program quality to youth purpose and from youth purpose to perceived program quality were stable across time (Models 4–5). Constraining within‐time covariances across waves resulted in a modest increase in *χ* ^2^; however, global fit indices remained acceptable and largely unchanged (Model 6). These results supported the selection of a fully constrained and more parsimonious final model.

Figure 1 displays the final selected CLPM. The autoregressive paths for both perceived program quality and youth purpose were statistically significant across all three waves (*p* < .001). Whereas the standardized coefficients were modest in magnitude (ranging from 0.23 to 0.27), their robust statistical significance demonstrates a reliable degree of continuity in youth's perceptions and purpose over time (Cohen, 2013).

**FIGURE 1:**
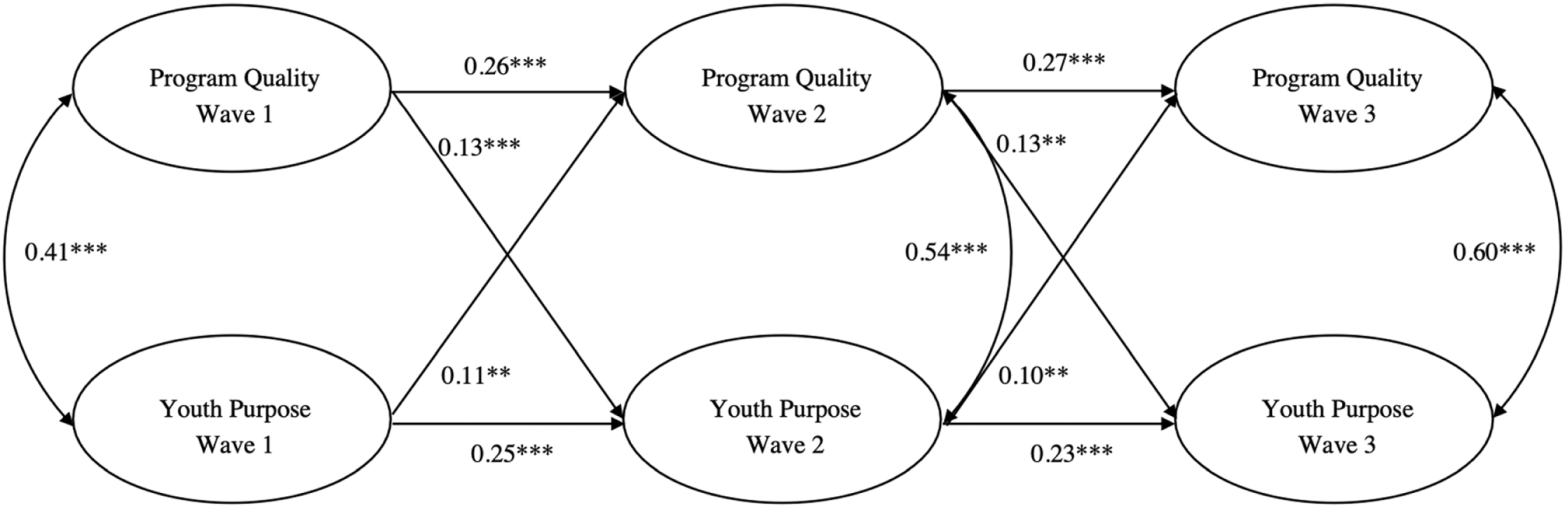
Visual depiction of the tested cross‐lagged panel model and standardized parameter estimates for relations between perceived program quality and youth purpose across three waves. Standardized coefficients are shown. Autoregressive paths, cross‐lagged paths, and within‐time correlations were estimated across Waves 1–3. *p < .05, **p < .01, ***p < .001.

Cross‐lagged associations between perceived program quality and youth purpose were positive and statistically significant, and stable across the three waves. Higher perceived program quality at one wave was positively associated with higher youth purpose at the subsequent wave (*β* = 0.13, *p* < .001). Conversely, youth purpose was also positively associated with subsequent perceptions of program quality (*β* = 0.10, 0.11, *p* < .01).

Within‐wave correlations between perceived program quality and youth purpose were positive and moderate at all three waves (*r* = .41, .60, *p* < .001), indicating that youth who perceived higher program quality also reported higher levels of purpose concurrently.

### Temporal precedence between perceived program quality and youth purpose

Temporal precedence between perceived program quality and youth purpose was examined by imposing equality constraints on the reciprocal cross‐lagged paths within the CLPM (Model 7). The model in which cross‐lagged paths from perceived program quality to youth purpose and from youth purpose to perceived program quality were constrained to be equal did not exhibit a significant decrease in model fit relative to the model without these constraints (Δ*χ* ^2^ = 3.451, Δ*df* = 1; ΔCFI = 0.000). This finding indicates that the magnitudes of the cross‐lagged effects did not differ by direction.

In other words, perceived program quality did not exert a stronger longitudinal influence on subsequent youth purpose than youth purpose exerted on subsequent perceptions of program quality. This pattern suggests that, across the three waves, neither construct served as a clear developmental driver of change in the other.

## DISCUSSION

Situated within the context of CI Study of PYD in Uganda, findings from the present study extend prior PYD research by clarifying how perceived program quality and youth purpose coact over time. By integrating rigorous longitudinal measurement with theory‐driven tests of developmental processes, this study contributes to the growing international literature on PYD and program effectiveness. It also provides empirical evidence relevant to practitioners and policymakers seeking to design and implement youth programs that foster purpose and thriving among youth in low‐resource contexts such as Uganda.

The present study sought to advance understanding of youth program effectiveness from a RDS perspective by examining longitudinal and reciprocal relations between perceived program quality and youth purpose among adolescents participating in CI programs in Uganda. Specifically, the study addressed two main goals: (1) investigating the longitudinal relations between perceived program quality, modeled as a higher‐order latent construct integrating the Big Three features, and youth purpose; and (2) to test temporal precedence by evaluating whether perceived program quality predicts subsequent youth purpose, whether youth purpose predicts perceived program quality, or whether these associations are reciprocal over time. By integrating rigorous longitudinal measurement with theory‐driven tests of person⇔context coactions, this study responds directly to calls for research that moves beyond cross‐sectional and unidirectional models of program influence (Lerner et al. 2026; Overton, 2015).

Whereas our theoretical framework is grounded in RDS concepts, which emphasize dynamic, coactions between individuals and their contexts across time and place, it is important to note that our analytic approach (CLPM) could not fully distinguish between within‐person and between‐person processes. Therefore, whereas our findings of bidirectional associations are conceptually consistent with the theoretical expectations of RDS, they reflect a combination of within‐ and between‐person reciprocal relations rather than directly demonstrating causality or the dynamic within‐person relations emphasized within RDS concepts. Future research utilizing approaches such as the Random‐Intercept Cross‐Lagged Panel Model (RI‐CLPM) is needed to explicitly disaggregate these within‐person processes and, as well, if there is sufficient power in future RDS‐based longitudinal data sets, statistical methods that can more closely align with theoretical ideas about dynamic coactions, such as dynamic SEM (McNeish & Hamaker, 2020) and state space grids (Hollenstein, 2007) should be consider for use.

Findings revealed autoregressive stability for both constructs, as well as positive and statistically significant reciprocal associations between perceived program quality and youth purpose across waves. Furthermore, tests of temporal precedence indicated that neither perceived program quality nor youth purpose emerged as a stronger longitudinal driver of change in the other, pointing instead to a pattern of mutual influence rather than unidirectional effects.

Consistent with a RDS perspective, findings indicate that perceived program quality and youth purpose are dynamically and reciprocally linked over time (Lerner, 2026; Overton, 2015). Higher perceived program quality predicted increases in subsequent youth purpose, suggesting that supportive, engaging, and developmentally responsive program contexts may create conditions that nurture youths' sense of meaning and direction (Damon et al., 2019). At the same time, youth purpose predicted later perceptions of program quality, indicating that purposive youth may engage more intentionally with program opportunities and interpret program features as more meaningful and supportive (Damon, 2025; Spencer, 2006). These bidirectional associations highlight youth as active agents in shaping their developmental contexts, rather than passive recipients of program inputs (Bornstein, 2019).

The absence of a dominant directional effect suggests that neither perceived program quality nor youth purpose functions as a stronger developmental driver in isolation. Instead, the equivalence of cross‐lagged effects aligns well with RDS perspective, which emphasizes mutually influential person⇔context processes coacting over time (Lerner, 2021, 2026; Overton, 2015). From this perspective, attempts to isolate one construct as the primary causal force may obscure the dynamic processes through which youth strengths and contextual supports co‐evolve over time.

In addition, conducting this study within the context of the CI PYD in Uganda extends the literature on youth purpose and program quality beyond predominantly U.S. samples, which have historically dominated developmental research (DeSouza, 2017; Hershberg et al., 2015). In low‐resource contexts, where youth often experience cumulative risk and constrained educational and economic opportunities (Culbreth et al., 2021), purpose may function as a particularly salient developmental asset (see Abbasi‐Asl, Yu, Hasse, et al., 2025) whereas the quality of developmental youth programs may play a critical role in shaping youths' developmental trajectories (see Abbasi‐Asl, Keces, Mackin, et al., 2025). Demonstrating reciprocal relations between youth purpose and perceived program quality in this setting provides empirical support for the cross‐context relevance of RDS processes, suggesting that mutually influential person⇔context dynamics operate across diverse cultural and structural environments.

Another key contribution of the present study is the use of a latent higher‐order construct to represent perceived program quality, integrating the Big Three features of effective youth programs: (1) positive and sustained adult–youth relationships, (2) life‐skill‐building activities, and (3) youth contribution and leadership opportunities, within a single longitudinal analytic framework. Rather than modeling these features as isolated dimensions, the present study treats perceived program quality as a shared latent context that captures youths' holistic experiences of program environments across time. This approach extends prior work that has typically examined the Big Three features separately (Tirrell et al., 2020, 2021).

### Limitations and future directions

Several limitations should be considered when interpreting the present findings. First, although CLPM allowed for tests of relations between perceived program quality and youth purpose, these models do not fully disentangle within‐person change from between‐person differences. Prior longitudinal research in Majority World contexts (e.g., South Africa, see Abbasi‐Asl, Keces, Lerner, et al., 2025), and specifically among Ugandan youth (Abbasi‐Asl et al., 2026; Abbasi‐Asl, Yu, Hasse, et al., 2025; Abbasi‐Asl, Yu, Tirrell, et al., 2025), has shown notable individual differences in character strengths and demonstrated that within‐person associations can differ substantially from between‐person averages, with distinct patterns of relations emerging for individual youth that may not be apparent in group‐level analyses (Abbasi‐Asl, Yu, Hasse, et al., 2025; Abbasi‐Asl, Yu, Tirrell, et al., 2025). Future research could employ approaches such as random‐intercept CLPM or intensive longitudinal designs to more precisely capture within‐person, coactional developmental processes over time.

Second, the study focused on a specific population, Ugandan adolescents participating in CI programs, which limits the generalizability of the findings. Although this context represents an important contribution to PYD research in low‐resource, majority world settings, the relatively homogeneous sample and programmatic focus constrain inferences to other youth populations within Uganda and beyond. Future studies should include more diverse samples, including youth involved in other PYD or character development programs, youth from additional majority world countries, and youth without prior program involvement to better evaluate the robustness and boundary conditions of observed person–context relations.

Finally, reliance on youth self‐reports for both perceived program quality and youth purpose introduces potential concerns related to common method variance, response‐style bias, and social desirability effects (Card, 2017; Clifton, 2020). Future work should triangulate findings using multiple methods and informants, such as observational assessments, qualitative interviews, and reports from peers, teachers, or caregivers, to strengthen the robustness of study design.

## CONCLUSIONS AND IMPLICATIONS

The present study advances understanding of youth program effectiveness by demonstrating that perceived program quality and youth purpose are dynamically and reciprocally related over time among adolescents participating in CI programs in Uganda. By establishing longitudinal measurement invariance and modeling perceived program quality as a higher‐order latent construct integrating the Big Three features of effective youth programs, this study provides robust evidence that youths' perceptions of program contexts can be meaningfully and reliably assessed across time. The observed stability and reciprocal associations point to the importance of examining youth development as a longitudinal process rather than as a series of isolated, cross‐sectional effects.

The findings offer strong empirical support for a RDS perspective and the model of dynamic person⇔context relations, which emphasize that development emerges through sustained, bidirectional coactions between individuals and their environments. Rather than identifying a single dominant causal pathway, the results indicate that program contexts and youth purpose mutually influence one another over time, highlighting youth as active contributors to their developmental contexts. This study extends prior PYD research by providing longitudinal evidence that aligns with core dynamic, RDS assumptions about plasticity, agency, and contextual embeddedness. Methodologically, this work contributes to the literature by supporting the use of a higher‐order latent representation of the Big Three as an integrated indicator of perceived program quality. This conceptualization captures youths' holistic experiences of program environments and offers a parsimonious yet theoretically grounded approach for evaluating program processes in longitudinal research.

From an applied perspective, the results have important implications for youth program structure and design, particularly in low‐resource settings similar to Uganda. Programs that intentionally cultivate supportive relationships, skill‐building opportunities, and meaningful youth engagement may not only foster youth purpose but also be perceived as increasingly effective by purposive youth over time. This reciprocal process suggests that program quality and youth strengths should be considered jointly in both program implementation and evaluation.

It should be noted that the positive linear association between perceived program quality and youth purpose found in the present research may have important cautionary implications for program implementation. Because this positive association means that youth entering with lower levels of purpose may subsequently perceive lower program quality, it also means that youth who already possess foundational levels of purpose may be more primed to recognize, engage with, and benefit from program resources. It may be, then, that youth experiencing difficulties establishing initial purpose might lack the readiness to connect with adult mentors or recognize skill‐building opportunities, leading them to perceive the program as lower quality. To prevent these vulnerable youth from being left behind, practitioners must avoid providing homogenous support to a diverse group of youth. Programs should actively assess youth needs upon entry and provide targeted, foundational scaffolding, such as pairing low‐purpose youth with highly trained mentors who can proactively build trust, to ensure the program is accessible and effective for those who need it most.

Collectively, these findings illuminate the importance of designing youth programs that attend to ongoing person–context coactions and support continued investment in theoretically grounded, longitudinal evaluations of PYD initiatives in Majority World contexts.

## AUTHOR CONTRIBUTIONS

**Ruoying Zhang:** Conceptualization; methodology; software; visualization; writing – original draft; writing – review and editing; data curation; formal analysis; validation. **Roya Abbasi‐Asl:** Conceptualization; methodology; validation; supervision; writing – review and editing; data curation. **Margaret Mackin:** Data curation; formal analysis; writing – review and editing. **Jacqueline V. Lerner:** Supervision; writing – review and editing; funding acquisition; validation; resources. **Richard M. Lerner:** Supervision; writing – review and editing; funding acquisition; validation; resources. **Alistair Sim:** Supervision. **Elizabeth M. Dowling:** Supervision; data curation; resources. **Jonathan M. Tirrell:** Data curation; supervision; resources; methodology. **Natasha Keces:** Data curation. **Alexa Hasse:** Data curation. **Kirsten Olander:** Data curation. **Kiana Douglas:** Project administration; resources; investigation. **Patience Kibbedi:** Project administration; resources; investigation. **Jane Wanyama:** Project administration; resources; investigation.

## FUNDING INFORMATION

The writing of this paper was supported in part by grants from Compassion International (CW2276870) and the Templeton World Charity Foundation (TWCF0516).

## CONFLICT OF INTEREST STATEMENT

The authors declare that there are no conflicts of interest.

## ETHICS STATEMENT

Ethical approval for this research was obtained from the Social, Behavioral, and Educational Research Institutional Review Board (Tufts University; IRB ID: 1606030). The research study and protocol were also approved by the Uganda National Council for Science and Technology (SS1110ES). All procedures performed in this study involving human participants were in accordance with the ethical standards of the institutional review board and Uganda National Council for Science and Technology.

## Supporting information


Appendix S1.

## Data Availability

The data are not publicly available due to privacy or ethical restrictions.
