# Supplementary material for: On the coactions between perceived program quality and youth purpose: Toward a model of dynamic person⇔context relations
Source: J Res Adolesc. 2026 Oct 6;36(4):e70277. doi: 10.1111/jora.70277 (PMC13639514; doi:10.1111/jora.70277)
Supplement: Supplementary file 1 [file JORA-36-0-s001.docx]

**Supplementary Appendix: Initial CFA Model Specification and Item Modification**

In our initial confirmatory factor analysis (CFA) of program quality using the Wave 1 dataset, we identified substantial semantic overlap between item adult7 ("Help you to gain the skills you need to succeed in life.") and item lern1 ("Learn skills that help you succeed in life."). The initial CFA yielded marginal model fit indices: χ²(132) = 406.88, *p* < .001; CFI = .895; TLI = .879; RMSEA = .059, 90% CI [.043, .052]; SRMR = .056. Modification indices indicated a strong need to free the residual covariance between adult7 and lern1 (Modification Index = 84.616, Expected Parameter Change = 0.517). Rather than retaining both items and correlating their residuals, we opted to remove adult7 due to redundancy, resulting in six remaining items in the “Positive and Sustained Adult–Youth Relationships” subscale. After removing adult7, the results demonstrated improved and acceptable model fit indices: χ²(116) = 224.653, *p* < .001; CFI = .955; TLI = .947; RMSEA = .039, 90% CI [.032, .047]; SRMR = .038. Consequently, this item was excluded from all further CFA, measurement invariance testing, and cross-lagged panel model (CLPM) analyses.

*Note:* These initial CFAs did not account for the nested data structure (TYPE = COMPLEX in Mplus were not utilized).
